# Role of Liquid-Phase Amount in Ceramization of Silicone Rubber Composites and Its Controlling

**DOI:** 10.3390/ma15103675

**Published:** 2022-05-20

**Authors:** Haibo Pang, Shiquan Zhang, Lei Pan, Suohui Yang, Jian Zhang, Minxian Shi, Zhixiong Huang, Junguo Li, Qiang Shen

**Affiliations:** State Key Laboratory of Advanced Technology for Materials Synthesis and Processing, Wuhan University of Technology, Wuhan 430070, China; haibopangjed@126.com (H.P.); zsq010608@163.com (S.Z.); pl1642895564@126.com (L.P.); yang177806@126.com (S.Y.); zhangjian178@whut.edu.cn (J.Z.); minxianshi@whut.edu.cn (M.S.); zhixiongh@whut.edu.cn (Z.H.); sqqf@263.net (Q.S.)

**Keywords:** B_2_O_3_, Al_2_O_3_, liquid phase, mechanical properties, ceramizable silicone rubber composites

## Abstract

The reliable mechanical properties of ceramizable silicone rubber composites during pyrolysis are necessary for their application in the fire-resistant fields. The effects of liquid-phase amount on the mechanical properties of silicone rubber composites are investigated. The results show a positive correlation between the liquid-phase amount and the flexural strength of the residual products pyrolysis below 800 °C. The nano-γ-Al_2_O_3_ in the fillers reacts with liquid B_2_O_3_ to form aluminum borate above 800 °C, which consumes the liquid phase and strengthens the residual products to a certain extent. Increasing the B_2_O_3_ addition and introducing nano-γ-Al_2_O_3_ can control the liquid-phase amount in the range of 15% to 30%, which makes the composites have better residual strength and support performance. The residual strength of composites pyrolysis at 500 °C to 1000 °C is higher than 2.50 MPa, and the maximum is up to 18.7 MPa at 1000 °C.

## 1. Introduction

Silicone rubber has a high pyrolysis temperature (higher than 300 °C) [1]. The main final pyrolysis product is amorphous SiO_2_ with oxidation resistance and insulation properties [2,3,4]. Therefore, silicone rubber has been widely used in fire-resistant fields.

Although the amorphous SiO_2_ produced by the pyrolysis of silicone rubber has no flammability, it has severe pulverization and poor mechanical properties [5]. Therefore, inorganic fillers need to be introduced into ceramizable silicon rubber composites to form a continuous ceramic body with a certain strength during the pyrolysis process [6,7]. Low melting point sintering additives (B_2_O_3_, glass frits, APP, etc.) are usually introduced into the composites to decrease the ceramization temperature. For example, Anyszka et al. carried out an investigation on the influence of B_2_O_3_ (melting point 450 °C) and boron-containing glass (melting point 780 °C) on microstructure and mechanical properties of silicone-basing composites after ceramization. The results show that more glassy phase binds pyrolysis products to form a solider ceramic bodies at 650°C, with the addition of B_2_O_3_ increased from 6.7 wt.% to 13.3 wt.% [8]. Mansouri et al. add glass frits with a 525 °C melting point into silicone rubber composites. The results show that the glass frits melt below the mica-silica eutectic temperature and combines with inorganic fillers and pyrolysis products to promote the formation of a ceramic. The flexural strength pyrolyzed at 600 °C, 800 °C, and 1000 °C were 0.88 MPa, 2.30 MPa, and 3.53 MPa, respectively [9]. Hu et al. carried out a study about the ceramifying process and mechanical properties of silicone rubber/ammonium polyphosphate/aluminum hydroxide/mica composites. The results show that the composites residues have a certain flexural strength pyrolyzed at 300 °C, 400 °C, 600 °C, 800 °C, and 1000 °C (2.54 MPa, 3.72 MPa, 3.46 MPa, 6.0 MPa, and 9.9 MPa, respectively) and good self-supporting performance [10]. Guo et al. report the influence of the glass frits content on the microstructure of silicone rubber composites. The results show that the flexural strength and impact strength of ceramic residue pyrolyzed at 1000 °C increases with the content of the glass frits. When the content of glass frits are up to 15 phr, the sufficient liquid phase appears to bind the mica and some pyrolysis products to form the stronger ceramic residue [11]. The sufficient liquid phase appearing at the range of 500 °C to 800 °C may also be helpful to improve the residual strength. Nevertheless, the added fillers are converted into liquid phase with decreasing viscosity with elevated temperatures, and then melt dripping behavior of the residues occurs, which negatively affects the ceramifying process [12]. Several papers confirm that low-temperature (500 °C~800 °C) residual strength can be improved to some extent by introducing low melting point glass frits, but the effect is not obvious. The excess liquid phase transformed from glass frits at high temperature is difficult to control, which limits the high-temperature support performance of the residual products. APP can make the composite self-supporting at high temperature, but it is hard to support a load. It is hoped to increase the liquid-phase amount during low-temperature pyrolysis to improve the residual strength. Still, the support performance of the residual products at high temperatures also needs to be guaranteed by decreasing the liquid phase. It is a challenge to improve the residual strength and support performance during the whole pyrolysis process.

In this paper, we increase the addition of B_2_O_3_ on the basis of others’ research. The B_2_O_3_ melting (450 °C) and B_2_O_3_-SiO_2_ eutectic (423 °C) increase the liquid phase to reinforce the residual products pyrolysed at low temperature. Meanwhile, nano-γ-Al_2_O_3_ is added to react with B_2_O_3_ consuming the excess liquid phase and forming the aluminum borate whisker at elevated temperatures (higher than 800 °C) to improve the support performance of residual products. The influence of liquid-phase amount on the residual strength of the composites is investigated.

## 2. Materials and Methods

### 2.1. Materials

Methyl vinyl silicone rubber (Zhonghao Chenguang Chemical Research Institute Co., Ltd., Zigong, China), fumed silica (KS-230T Nanjing Zaisheng New Material Co., Ltd., Nanjing, China), 2,4-Dichlorobenzoyl peroxide (DCBP) (Dongguan Hengcheng silicone Co., Ltd., Dongguan, China), boric oxide (Shanghai Aladdin Biochemical Technology Co., Ltd., Shanghai, China), mica (Shandong Yusuo Chemical Technology Co., Ltd., Linyi, China), nano-γ-Al_2_O_3_ (10 nm Guangzhou Nano Chemical Technology Co., Ltd., Guangzhou, China), and silicon dioxide (Sinopharm Chemical Reagent Co., Ltd., Shanghai, China) are used in the experiments.

### 2.2. Preparation

Silicone rubber, fumed silica, SiO_2_, mica, B_2_O_3_, nano-γ-Al_2_O_3_, and DCBP are mixed evenly in an open mixer, and then vulcanized by a plate vulcanizing machine. The vulcanization pressure is 10 MPa, the vulcanization temperature is 130 °C, and the vulcanization time is 10 min. After that, an oven is used for secondary vulcanization. The vulcanization temperature is 130 °C, and the vulcanization time is 4 h. The formulations of ceramizable silicone rubber composites are listed in Table 1. The sample symbols are labeled as B_12_A_36_, B_24_A_36_, B_36_A_36_, B_48_A_36_, B_60_A_36_, and B_48_A_0_.

### 2.3. Sample Pyrolysis

The rectangular samples are performed in a muffle furnace. It is heated up to the corresponding temperature (500 °C, 600 °C, 700 °C, 800 °C, 900 °C, 1000 °C) at a heating rate of 10 °C min^−1^ under air atmosphere, held for 30 min, and then cooled to room temperature.

### 2.4. Characterization

The phase analysis of residual products is characterized by X-ray diffraction with an X-ray diffractometer (Empyrean, Eindhoven, Netherlands). The grinded residual products are corroded with 1 mol L^−1^ hydrochloric acid for 24 h. The contents of aluminum (C_Al_) and boron (C_B_) in corrosion solution are analyzed by an Inductively Coupled Plasma-Optical Emission Spectrometer (ICP-OES Prodigy/7, Hudson, MA, USA). The mass ratio of B_2_O_3_ or nano-γ-Al_2_O_3_ participating in the reaction is calculated by:

$$R=\left(1-\frac{C_{X}\times V\times M_{X_{2}O_{3}}}{2M_{X}\times m}\right)\times 100\%$$

*M* is the relative molecular (atomic) mass, *X* is Al or B, *m* is the sampling mass, *V* is the volume of corrosive solution. The three-point flexural strength of the residual products is tested by an Instron Universal Testing Machine. The loading speed is 2 mm min^−1^, the sample size is 80 mm × 10 mm × 4 mm, and the span is 64 mm. A field emission scanning electron microscope (Quanta + FEG250, FEI Co., Hillsboro, OR, USA) is used to analyze the fracture morphology of samples after flexural strength test. The apparent porosity and bulk density of the pyrolysis samples are measured by Archimedes drainage method. The apparent porosity ($P_{a}$) is calculated by:

$$P_{a}=\frac{m_{2}-m_{0}}{m_{2}-m_{1}}\times 100\%$$

The bulk density ($\rho_{b}$) is calculated by:

$$\rho_{b}=\frac{m_{0}}{m_{2}-m_{1}}\times \rho_{Ethanol}$$

Dry weight, buoyant weight, and wet weight are represented by *m*_0_, *m*_1_, and *m*_2_. The support performance test of residual products at high temperature is placing the samples (80 mm × 10 mm × 4 mm) on two support points with a span (*L* = 60 mm), and the middle of the samples place a load twice the weight of the sample. The samples are heated up to the same temperature as the pyrolysis at a heating rate of 10 °C min^−1^ in a muff furnace, and held for 30 min. The distance (*d*) from the lowest point to the original position is measured. The bending angle (θ) is calculated by:

$$\tan\theta =\frac{2d}{L}$$

## 3. Results and Discussion

### 3.1. Microstructure of Residual Products

**Figure 1:**
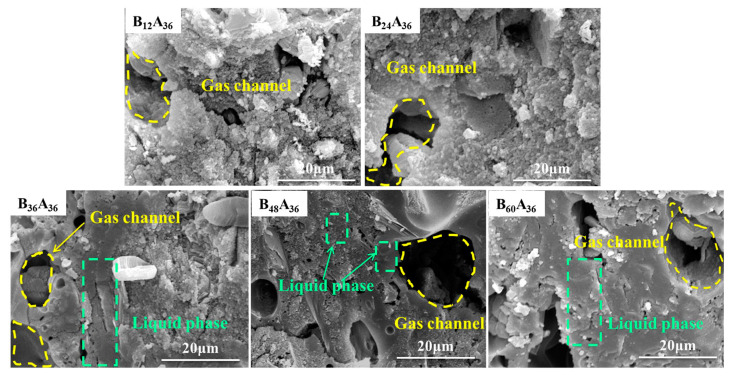
SEM images of fracture of samples with different addition of B_2_O_3_ pyrolyzed at 600 °C.

The samples with different B_2_O_3_ addition are pyrolyzed at 600 °C, and the SEM images of samples’ fracture are shown in Figure 1. It shows that the fracture is loose in sample B_12_A_36_ and sample B_24_A_36_. No liquid phase enrichment zone can be seen. In sample B_36_A_36_, there is some liquid phase enrichment zones that appear around the gas channel. The ceramic phase around the channel is more compact than other areas. The liquid enrichment zones mainly appear around the gas channel, and also distribute sporadically in other areas in sample B_48_A_36_. The fracture is smooth, and the liquid phase spreads throughout the ceramic phase in sample B_60_A_36_. That makes the compactness of other areas in the ceramic phase similar to that around the gas channel. It illustrates that the compactness of the ceramic phase improves with the addition of B_2_O_3_.

**Figure 2:**
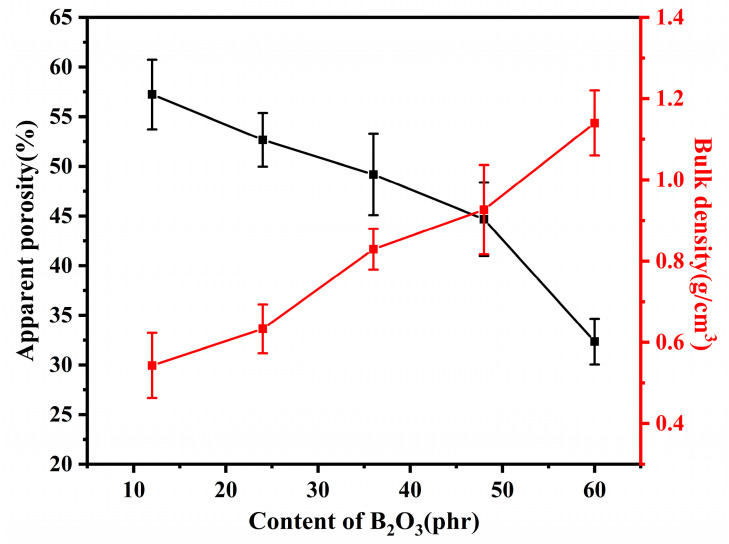
Apparent porosity and bulk density of samples with different addition of B_2_O_3_ pyrolyzed at 600 °C.

Figure 2 shows the apparent porosity and bulk density of samples with different addition of B_2_O_3_ pyrolyzed at 600 °C. It is shown that the apparent porosity decreased with the addition of B_2_O_3_ in the composites, and the bulk density improved with the addition of B_2_O_3_. Therefore, increasing the addition of B_2_O_3_ can promote the densification of the ceramic phase.

**Figure 3:**
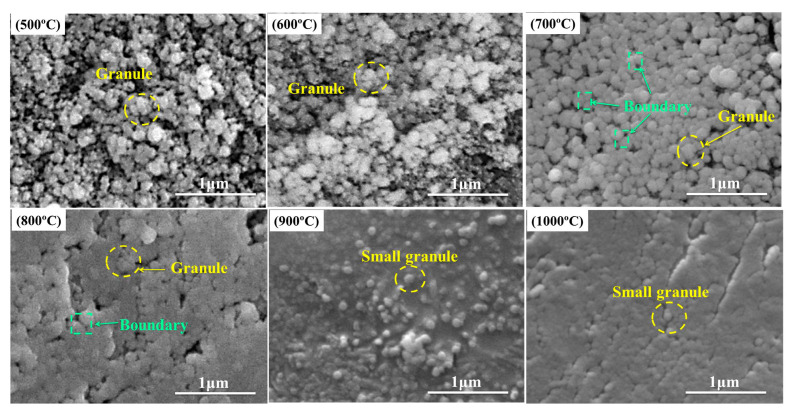
SEM images of fracture of B_48_A_36_ pyrolyzed at different temperatures.

Sample B_48_A_36_ are pyrolyzed at different temperatures, and the SEM images of fracture are shown in Figure 3. It shows that the fracture surface is very coarse at 500 °C. There are many irregular granules at the fracture. At 600 °C, the granules at the fracture are regular spherical. The number of granules increases significantly compared to 500 °C. The granules connect and aggregate into larger granules. At 700 °C, the granules transform into spheroids with smooth surfaces. Granules form connections with each other. The continuous ceramic structure is formed. Granules are still integrity and clear boundary. At 800 °C, most granule boundaries disappear. The ceramic phase is more compact than that at 700 °C. At 900 °C, granule boundaries and pores at the fracture disappear entirely. Small granules can be observed, embedded in the compact ceramic phase. The small granules are entirely spherical. The small particles embedded in the ceramic phase disappear at 1000 °C, and the compactness of the ceramic phase is further improved. Therefore, the compactness of the ceramic phase improves with the temperatures.

### 3.2. Phase analysis

**Figure 4:**
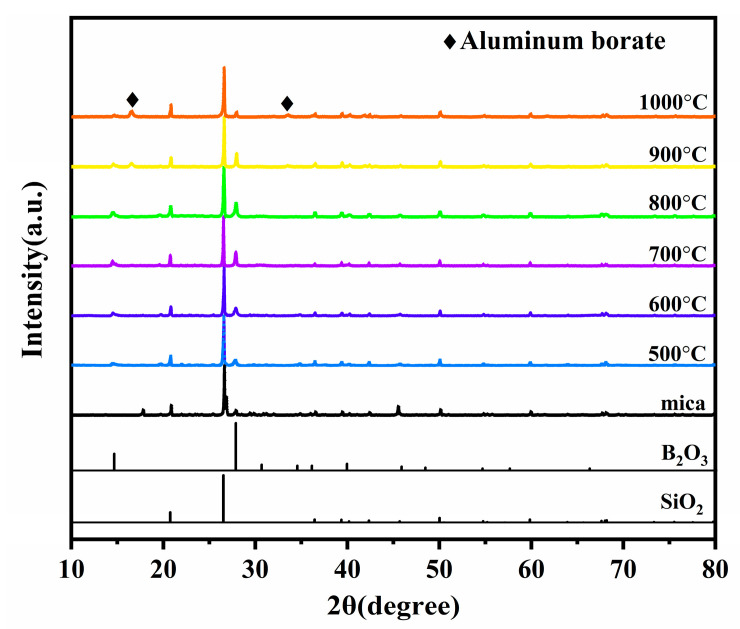
XRD patterns of sample B_48_A_36_ pyrolyzed at different temperatures.

Figure 4 shows the XRD patterns of sample B_48_A_36_ pyrolyzed at different temperatures. It is shown that the main phases of the residual products below 800 °C are SiO_2_ (PDF 46-1405) and mica. The diffraction peaks of aluminum borate appear when sample B_48_A_36_ pyrolyzed at 900 °C and 1000 °C. The consumption of B_2_O_3_ and nano-γ-Al_2_O_3_ during the ceramization process is shown in Table 2. It shows that the consumption of B_2_O_3_ is 20% and 21% of the addition, and that of nano-γ-Al_2_O_3_ both are 89% of the addition at 900 °C and 1000 °C. The measured consumption mass ratio of B_2_O_3_ and nano-γ-Al_2_O_3_ deviates from that which forms a single-phase aluminum borate. Therefore, the actual aluminum borate formed is a two-phase mixture [13]. The content of the two phases in the mixture is similar. 2Al_2_O_3_·B_2_O_3_ is 85%, and 9Al_2_O_3_·2B_2_O_3_ is 15%. The reactions of forming aluminum borate are given by:

2Al_2_O_3_ (s) + B_2_O_3_ (l) = 2Al_2_O_3_·B_2_O_3_ (s) (1)

9Al_2_O_3_ (s) + 2B_2_O_3_ (l) = 9Al_2_O_3_·2B_2_O_3_ (s) (2)

**Figure 5:**
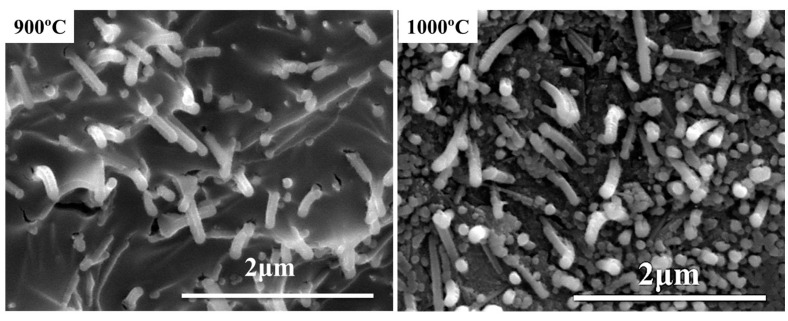
SEM images of aluminum borate whisker at the fracture of sample B_48_A_36_ pyrolyzed at different temperatures.

It illustrates that nano-γ-Al_2_O_3_ can react with the liquid-state B_2_O_3_ to form the solid-state aluminum borate above 800 °C. There are aluminum borate whiskers [13,14] in some regions of the ceramic phase. The SEM images of the aluminum borate whisker around the wall of the channels are shown in Figure 5.

### 3.3. Mechanical Properties

**Figure 6:**
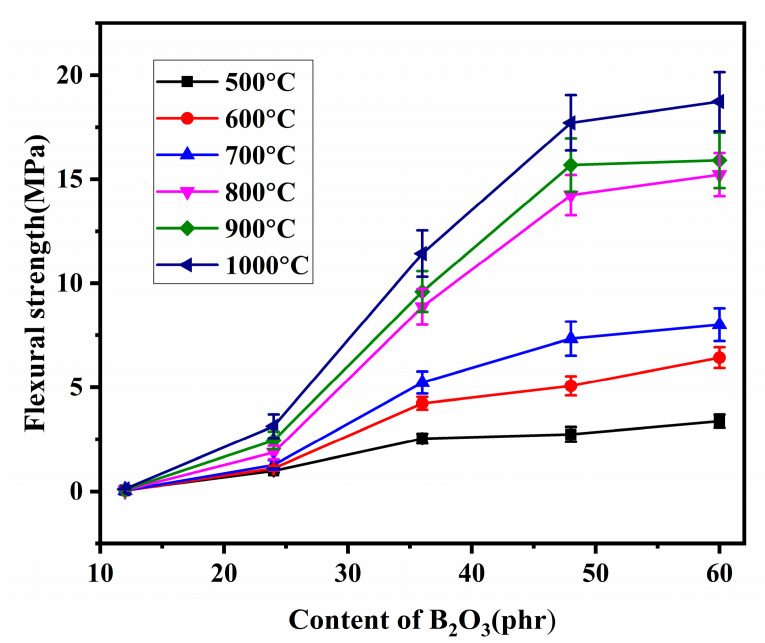
Flexural strength of residual products with different addition of B_2_O_3_.

The flexural strength of residual products with different B_2_O_3_ addition is shown in Figure 6. It shows that the flexural strength of residual products pyrolyzed at the same temperatures improves with the B_2_O_3_ addition, especially when the pyrolysis temperatures are in the range of 800 °C~1000 °C and the addition of B_2_O_3_ is less than 48 phr. However, the effect of further addition of B_2_O_3_ on the flexural strength is not significant.

In addition, the flexural strength of B_12_A_36_ and B_24_A_36_ pyrolyzed at different temperatures are in the range of 0.06 MPa~0.12 MPa and 0.99 MPa~3.1 MPa. In contrast, B_36_A_36_, B_48_A_36_, and B_60_A_36_ are in the range of 2.50 MPa~11.4 MPa, 2.74 MPa~17.7 MPa, and 3.38 MPa~18.7 MPa, respectively. The flexural strength of the residual products improves with the pyrolysis temperatures. This result is more pronounced when the B_2_O_3_ is added in the range of 36 phr~60 phr. When the addition of B_2_O_3_ is higher than 36 phr, there is a sharp improvement of flexural strength from 700 °C to 800 °C, and the flexural strength increases slightly above 800 °C.

**Figure 7:**
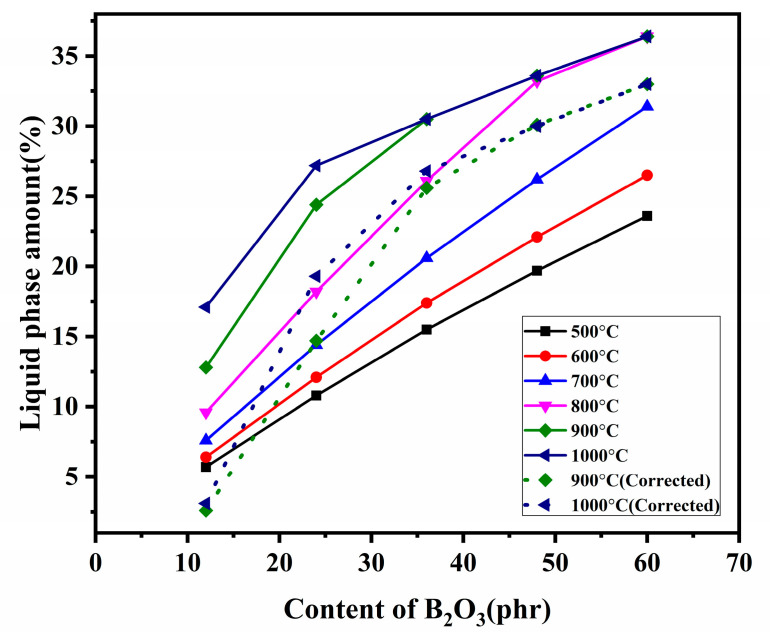
Liquid-phase amount in the composites with different addition of B_2_O_3_.

The liquid-phase amount (mass percent) in the composites calculated by the B_2_O_3_-SiO_2_ phase diagram is shown in Figure 7. The sources of SiO_2_ in the liquid phase are SiO_2_ in the fillers, fumed silica, and amorphous silica (pyrolysis products of silicone rubber) remained after the pyrolysis of composites. The liquid-phase amount represented by the solid lines is calculated according to the addition of B_2_O_3_. As a result of the formation of aluminum borate consuming the B_2_O_3_, the corrected content of B_2_O_3_ in the liquid phase at 900 °C and 1000 °C are the addition amount minus the reaction amount. Thus, the corrected liquid-phase amount in the composites is the dotted line.

In combination with Figure 6 and Figure 7, the improvement trend of the flexural strength is highly consistent with the increasing trend of the liquid-phase amount in the range of 500 °C~800 °C. It is easy to see from the microstructure in Figure 1 that increasing the liquid phase is beneficial to form a more compact structure of the residual products resulting in the mechanical properties [11]. The positive correlation between flexural strength and liquid-phase amount is further proved. The flexural strength of the residual products is higher than 2.50 MPa when the liquid-phase amount is higher than 15%.

When the pyrolysis temperature is higher than 800 °C, the liquid-phase amount is decreased due to the formation of aluminum borate. However, the compactness of the ceramic phase is not affected by that at 900 °C and 1000 °C. The continuous ceramic phase has been formed at 800 °C, as shown in Figure 3, and the formation of aluminum borate whisker that is usually used as reinforce and refractory [15,16,17] reinforces the ceramic phase. Therefore, the flexural strength does not weaken at 900 °C and 1000 °C but improves. In a word, the strengthening effect of aluminum borate whisker and the bonding effect of the liquid phase together affect the flexural strength of residual products pyrolyzed above 800 °C.

In addition, when the pyrolysis temperature is higher than 800 °C and the addition of B_2_O_3_ is higher than 48 phr, the effect of increasing B_2_O_3_ to the liquid amount on flexural strength is not obvious. As can be seen from Figure 3, the liquid-phase amount (33%) at 800 °C is enough to form a continuous ceramic phase. Therefore, further increasing the liquid-phase amount to improve flexural strength is meaningless.

**Figure 8:**
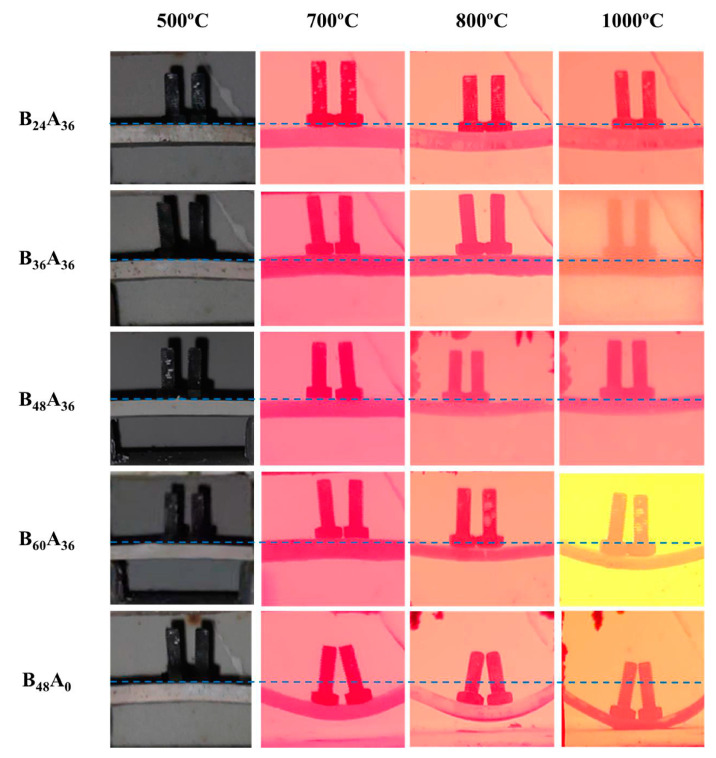
Support performance of residual products.

As a result of the flexural strength of B_12_A_36_ pyrolyzed at different temperatures less than 1 MPa, it is too weak to support twice their weight. Therefore, the support performance is not able to be measured. The support performance of other residual products is shown in Figure 8, and the bending angle is listed in Table 3. The smaller the bending angle is, the better support performance of the residual products has. It shows that all the residual products could support twice their weight at 500 °C without deformation. Although B_48_A_0_ has a bending angle of 25°, others also could support the load at 700 °C without deformation. At 800 °C, the bending angle of B_24_A_36_ is 10°. The support performance of B_24_A_36_ decreases due to the bad flexural strength. B_36_A_36_ remains unchanged. B_48_A_36_ and B_60_A_36_ are 12° and 14°, respectively. The support performance decreases with the addition of B_2_O_3_. The bending angle of B_48_A_0_ further increases to 31°. When the temperature is up to 1000 °C, the lowest point of the B_48_A_0_ with the bending angle of 40° touches the furnace and loses support capacity. B_24_A_36_, B_36_A_36_, and B_48_A_36_ remain unchanged. The bending angle of B_60_A_36_ is up to 20°. The decrease of support performance at 1000 °C is more obvious with the increase of B_2_O_3_. It is inferred that the residual products of samples with the addition of B_2_O_3_ less than 48 phr (include 48 phr) have good support performance. The further addition of B_2_O_3_ causes the decreasing of support performance due to the excess liquid phase. Compared with sample B_48_A_0_, B_48_A_36_ has better support performance. Therefore, the addition of nano-γ-Al_2_O_3_ could significantly improve the support performance of the residual products. According to Figure 4 and Figure 5, and Table 2, the liquid phase is adjusted by forming aluminum borate to a certain extent. The aluminum borate whisker also reinforces the residual products at the same time. It is the reason that the addition of nano-γ-Al_2_O_3_ improves the support performance. The corrected liquid-phase amount of B_48_A_36_ and B_60_A_36_ at 1000 °C is 30% and 33%. The support performance of B_48_A_36_ is better than that of B_60_A_36_ at 1000 °C. Therefore, the introducing of nano-γ-Al_2_O_3_ can only guarantee the support performance of residual products in a certain range of liquid phase, beyond which the support performance decreases.

In summary, increasing the liquid phase is helpful to improve the flexural strength of residual products. However, the excess liquid phase is harmful to the support performance at elevated temperatures. According to Figure 6, Figure 7 and Figure 8, when the liquid phase is higher than 15%, the composites pyrolyzed at different temperatures have good flexural strength. The liquid phase is higher than 30%, and the support performance of residual products at elevated temperatures decreases significantly. It illustrates that the liquid phase should be controlled at the range of 15% to 30% to guarantee reliable mechanical properties during the pyrolysis process. By increasing the addition of B_2_O_3_ and introducing nano-γ-Al_2_O_3_, the liquid phase amount in the composites is controlled within this range during the pyrolysis process, realizing the good residual strength and high-temperature support performance.

## 4. Conclusions

In combination with the B_2_O_3_-SiO_2_ phase diagram, the liquid phase increases with the addition of B_2_O_3_ and the pyrolysis temperature. The flexural strength of residual products increases with the liquid phase. The liquid-phase amount is the main factor affecting the flexural strength of the residual products pyrolyzed below 800 °C. Nano-γ-Al_2_O_3_ can improve the support performance of residual products above 800 °C. The liquid-phase amount is decreased by forming aluminum borate at elevated temperatures. The formed aluminum borate whiskers act as reinforcement, cooperating with liquid-phase amount to affect the flexural strength and the support performance of the residual products above 800 °C. The liquid phase is controlled in the range of 15% to 30% to have good flexural strength and support performance.

## Figures and Tables

**Table 1:** Formulations of ceramizable silicone rubber composites.

	Compositions (phr)	Silicone Rubber	SiO_2_	Mica	B_2_O_3_	Nano-γ-Al_2_O_3_	Fumed Silica	DCBP
Samples								
B_12_A_36_		100	24	36	12	36	30	2
B_24_A_36_		100	24	36	24	36	30	2
B_36_A_36_		100	24	36	36	36	30	2
B_48_A_36_		100	24	36	48	36	30	2
B_60_A_36_		100	24	36	60	36	30	2
B_48_A_0_		100	24	36	48	0	30	2

**Table 2:** Consumption of B_2_O_3_ and nano-γ-Al_2_O_3_ during ceramization process.

Temperature (°C)	Consumption of Nano-γ-Al_2_O_3_ (%)	Consumption of B_2_O_3_ (%)
900	89	20
1000	89	21

**Table 3:** The bending angle of residual products with different addition of B_2_O_3_.

Temperature (°C)	Bending Angle (°)				
	B_24_A_36_	B_36_A_36_	B_48_A_36_	B_60_A_36_	B_48_A_0_
500	-	-	-	-	-
700	-	-	-	-	25
800	10	-	12	14	31
1000	10	-	12	20	40

## Data Availability

The data reported in this article can be provided by corresponding author (Junguo Li) through reasonable requests.
